# Optimized Hepatitis C Virus (HCV) E2 Glycoproteins and their Immunogenicity in Combination with MVA-HCV

**DOI:** 10.3390/vaccines8030440

**Published:** 2020-08-05

**Authors:** María Q. Marín, Kwinten Sliepen, Juan García-Arriaza, Sylvie M. Koekkoek, Patricia Pérez, Carlos Óscar S. Sorzano, Carmen E. Gómez, Rogier W. Sanders, Mariano Esteban

**Affiliations:** 1 Department of Molecular and Cellular Biology, Centro Nacional de Biotecnología (CNB), Consejo Superior de Investigaciones Científicas (CSIC), 28049 Madrid, Spain; mquiros@cnb.csic.es (M.Q.M.); pperez@cnb.csic.es (P.P.); cegomez@cnb.csic.es (C.E.G.); 2 Department of Medical Microbiology, Amsterdam Infection & Immunity Institute, Amsterdam UMC, University of Amsterdam, 1105AZ Amsterdam, The Netherlands; k.h.sliepen@amsterdamumc.nl (K.S.); s.m.koekkoek@amsterdamumc.nl (S.M.K.); r.w.sanders@amsterdamumc.nl (R.W.S.); 3 Biocomputing Unit, Centro Nacional de Biotecnología (CNB), Consejo Superior de Investigaciones Científicas (CSIC), 28049 Madrid, Spain; coss@cnb.csic.es; 4 Department of Microbiology and Immunology, Weill Medical College of Cornell University, New York, NY 10065, USA

**Keywords:** HCV, E2 protein, cysteines, disulfide bonds, MVA, vaccine, mice, immune responses, CD4 and CD8 T cells, antibodies

## Abstract

Hepatitis C virus (HCV) represents a major global health challenge and an efficient vaccine is urgently needed. Many HCV vaccination strategies employ recombinant versions of the viral E2 glycoprotein. However, recombinant E2 readily forms disulfide-bonded aggregates that might not be optimally suited for vaccines. Therefore, we have designed an E2 protein in which we strategically changed eight cysteines to alanines (E2.C8A). E2.C8A formed predominantly monomers and virtually no aggregates. Furthermore, E2.C8A also interacted more efficiently with broadly neutralizing antibodies than conventional E2. We used mice to evaluate different prime/boost immunization strategies involving a modified vaccinia virus Ankara (MVA) expressing the nearly full-length genome of HCV (MVA-HCV) in combination with either the E2 aggregates or the E2.C8A monomers. The combined MVA-HCV/E2 aggregates prime/boost strategy markedly enhanced HCV-specific effector memory CD4^+^ T cell responses and antibody levels compared to MVA-HCV/MVA-HCV. Moreover, the aggregated form of E2 induced higher levels of anti-E2 antibodies in vaccinated mice than E2.C8A monomers. These antibodies were cross-reactive and mainly of the IgG1 isotype. Our findings revealed how two E2 viral proteins that differ in their capacity to form aggregates are able to enhance to different extent the HCV-specific cellular and humoral immune responses, either alone or in combination with MVA-HCV. These combined protocols of MVA-HCV/E2 could serve as a basis for the development of a more effective HCV vaccine.

## 1. Introduction

Hepatitis C virus (HCV) is a mayor public health problem, with an estimated 71 million people chronically infected and approximately 400,000 annual deaths. The World Health Organization (WHO) adopted in 2016 the first-ever global hepatitis strategy, setting 2030 as a deadline to eliminate HCV as a public health threat by reducing new infections by 90% and mortality by 65% [1]. However, the road towards these goals will be very difficult, since 80% of the 71 million infected people are unaware of their infection status and unknowingly spread the virus. Without an effective vaccine, it will be necessary to greatly improve screening programs, surveillance, HCV tests, infection control measures, and rapidly scale up the coverage of HCV treatment. Assuming these interventions would be successful, the total costs of such an effort are around US$ 11.9 billion for the period 2016-21 alone [2]. Vaccination is a proven method for infection prevention and an effective, accessible, and affordable vaccine is probably needed to eliminate HCV as a health threat by 2030 [1].

HCV is a rapidly evolving virus and it is highly sequence diverse. Nevertheless, approximately 30% of infected individuals can spontaneously clear the virus and acquire immunity that sometimes protects against reinfections with heterologous HCV genotypes [3,4,5,6,7,8,9], suggesting that a vaccine able to induce similar immune responses might protect against HCV infection. The main roadblocks for the development of effective HCV vaccines are the lack of an appropriate animal model, the HCV diversity (with up to 7 different genotypes and 35% sequence diversity among them) and that correlates of protection are not well-established [10,11]. However, it is widely accepted that a highly effective vaccine should elicit strong, broad and polyfunctional T cell responses together with broadly neutralizing antibodies (bNAbs) that neutralize most HCV genotypes [11,12,13,14]. The importance of memory T cells for protection against chronic disease has been shown by studies in chimpanzees, where both CD4^+^ and CD8^+^ T cells were needed to prevent HCV reinfection [15,16], while increasing evidence points to the key role of bNAbs in the prevention, control and even abrogation of HCV infection [8,17,18,19,20,21,22]. While T cell responses are usually directed against HCV nonstructural proteins (NS2, NS3, NS4, and NS5), antibody responses are mainly reactive against HCV envelope glycoproteins E1 and E2. 

We have previously described a poxvirus vaccine candidate against HCV based on the Modified Vaccinia Virus Ankara (MVA) that expresses the nearly full-length HCV genome from genotype 1a, strain H77 (termed MVA-HCV) [23]. In immunized mice, MVA-HCV elicited strong, broad, and polyfunctional HCV-specific CD8^+^ T cells [23,24]. Furthermore, an heterologous prime/boost immunization protocol involving a prime with DNA-based vaccines consisting of alphavirus DNA-launched replicons (DREP) followed by a boost with MVA-HCV, elicited potent HCV-specific CD8^+^ T cell responses, together with broad and polyfunctional HCV-specific CD4^+^ T cells [25]. However, these immunization protocols induced low antibody titers to HCV antigens, and more optimized protocols are needed to boost the humoral responses.

All neutralizing HCV antibodies target the heavily glycosylated E1E2 protein complex. E1E2 is essential for binding to and entry of HCV to hepatocytes [26]. The E2 subunit mainly interacts with CD81 and scavenger receptor class B 1, while the subunit E1 contains the putative fusion peptide. However, it is still unknown whether E1E2 is organized as one heterodimer or a trimer of E1E2 heterodimers [26,27,28]. The majority of bNAbs target the E2 subunit, while only a few bNAbs against E1 have been isolated [29,30,31,32]. Soluble recombinant versions of the ectodomain of E2 maintain receptor-binding properties and are recognized by most anti-E2 bNAbs, suggesting that recombinant E2 (partly) retains its conformation [33,34,35]. 

The sequence of E2 is highly variable, but the 18 cysteine residues in E2 are extremely conserved among all HCV genotypes, because they play an important role in the structure and function of E2 [36]. However, these cysteines form a heterologous disulfide bridge network that causes aggregation, imposing important restrictions in functional studies and in the elucidation of the three-dimensional structure of E2. This is illustrated by the significant differences in the location of the disulfide bonds reported in the crystal structures of E2, showing that the nature of experiments also influences disulfide bond formation [36,37,38,39,40]. In conclusion, the disulfide bonds differ between E2 protein constructs and their functional role in E1E2 is still undefined. 

Therefore, in the present study, we rationally designed an E2 protein lacking eight cysteines (E2.C8A). The resulting E2.C8A formed mostly monomers instead of aggregates and interacted efficiently with anti-E2 bNAbs. Lastly, we studied the immunogenicity of E2 and E2.C8A in vivo by immunizing mice with these proteins alone or in combination with an MVA-HCV priming immunization. The results of this study inform the design of optimized E2 immunogens and lays out possible vaccination strategies that are aimed at obtaining more balanced T and B cell responses against HCV.

## 2. Materials and Methods

### 2.1. Construction of Plasmids Containing HCV E2 Proteins

The HCV construct used to generate all E2 variants was based on a variant of the genotype 1a H77 strain reported in genbank# ABN11232.1 (amino acids 384–659) and genbank# AF009606.1 (amino acids 660–715). E2_MPER_ and E2.C8A_MPER_ encompass the complete E2 ectomain (amino acids 384–715; standard H77 numbering [41], while the recombinant E2 and E2.C8A encompass the ectodomain of E2, without the C-terminal membrane-proximal external region (MPER) (amino acids 384–698). The E2_MPER_.C8A and E2.C8A contain the following eight cysteine-to-alanine mutations: C452A, C486A, C569A, C581A, C585A, C597A, C652A, and C677A. The codon-optimized sequences of these E2 sequences (Genscript, Leiden, the Netherlands) were cloned into the mammalian expression plasmid pPPI4, as previously described [42]. 

### 2.2. E2 Protein Expression

The E2 proteins were transiently expressed in adherent human embryonic kidney (HEK) 293T or 293F cells as previously described [43,44]. HEK-293T cells were maintained in Dulbecco’s Modified Eagle Medium (DMEM) supplemented with 10% fetal calf serum (FCS, Gibco-Life Technologies, Carlsbad, CA, USA), penicillin (100 U/mL, ThermoFisher Scientific, Waltham, MA, USA) and streptomycin (100 μg/mL, ThermoFisher Scientific, Waltham, MA, USA), while HEK-293F cells were maintained in FreeStyle media (Life Technologies, Carlsbad, CA, USA) without antibiotics.

For protein expression at a small scale, 5.5 × 10^4^ HEK-293T cells/mL were seeded in a 6-well plate. The next day, when cells reached 60–70% confluency, they were transfected with 5 μg of plasmid and polyethyleneimine (PEImax 1 mg/mL, Polysciences, Warrington, PA, USA) as a transfection agent in OPTI-MEM (Gibco-Life Technologies, Carlsbad, CA, USA) as previously described [45]. The supernatants were harvested 48 h after transfection and centrifuged to eliminate any residual cell at 1500 rpm for 5 min. 

For large-scale E2 protein purification, 0.8–1.2 × 10^6^ HEK-293F cells/mL were transfected with PEImax and 300 µg of plasmid and cells were cultured for 6 days at 37 ℃, with 8% CO_2_ and a rotation speed of 125 rpm. Then, supernatants were obtained and we proceeded to protein purification. 

### 2.3. E2 Protein Purification

E2 proteins were purified from transfected supernatants of HEK-293F cells by affinity chromatography using Strep-Tactin^®^ XT (IBA Life Sciences, Göttingen, Germany) columns and following manufacturer’s recommendations. Briefly, supernatants were filtered through 0.2 µm filters and passed through the column at an approximate speed of 0.5 mL/min overnight. After two washing steps, bound E2 protein was eluted and concentrated in a Vivaspin 6 10 kDa cut-off filter (Sartorius, Göttingen, Germany) and left in the final buffer 20 mM Tris-HCl pH 8, 150 mM NaCl. Next, proteins were further fractionated by size-exclusion chromatography (SEC). The protein concentration was determined using Nanodrop with the theoretical extinction coefficient calculated via Expasy (ProtParam tool): E2 = 84.5 and E2.C8A = 85.

### 2.4. Neutralization Assays

To study neutralizing responses we first purified polyclonal IgG from the mice sera to reduce non-specific viral inhibitory effects, essentially as described [46]. The purified IgG samples were buffer exchanged into PBS using Vivaspin6 10kDa filters (Sartorius, Göttingen, Germany). Next, we performed neutralization assays using HCV pseudo particles (HCVpp) essentially as described before [6]. Briefly, we incubated serially diluted polyclonal mouse IgG with H77 (Genbank# AF009606) HCVpp for 1 h at 37 °C followed by spin-oculation (2000× *g* for 45 min) onto Huh-7 cells that were seeded at 50% confluency the day before. After three days, the Huh-7 cells were lysed using Bright-GloLuciferase Assay System (Promega Corporation, Madison, WI, USA). Neutralization was determined by calculating the relative luciferase activity compared to no virus (set at 100% neutralization) and virus only (set at 0% neutralization) controls. Experiments were performed once or twice independently and always in triplicate.

### 2.5. ELISA Assays

To study the antigenic properties of the produced E2 proteins from HEK-293T cell supernatants, microloan-600 96-well, half area plates (Greiner Bio-One, Kremsmünster, Austria) were coated overnight with *Galanthus nivalis* lectin (GNL) (Vector Laboratories, Burlingame, CA, USA; 20 μg/mL) in coating buffer at room temperature. Next day, plates were washed with Tris-buffered saline (TBS) and blocked with 2% casein-TBS 2% (ThermoFisher Scientific, Waltham, MA, USA) for 30 min at room temperature. Then, plates were washed again and 100 µL of supernatants were added to plates and incubated for 2 h at room temperature. After supernatant incubation, plates were washed twice with TBS and 100 µL of monoclonal antibodies were added: anti-Strep-tagII (THE^TM^ NWSHPQFEK-Tag Antibody (Genscript, Leiden, the Netherlands), 1 µg/mL), AT12-009 (3 µg/mL), AT12-011 (3 µg/mL), AP33 (0.5 µg/mL), HC84.26 (10 µg/mL), and CD81-LeL (3 µg/mL), diluted in 2% casein-TBS and incubated on the plate for 2 h at room temperature. After antibody incubation, plates were washed twice with TBS and 100 µL of secondary antibodies were added (Horseradish peroxidase (HRP)-labeled goat anti-human or anti-mouse IgG) diluted in 2% casein-TBS followed by 1 h of incubation at room temperature. After incubation, plates were washed 4 times with TBS-0.05% Tween20 and one last time with TBS before color development. Colorimetric detection was performed using a solution containing 1% 3,3’,5,5’ tetramethylbenzidine (TMB, Sigma-Aldrich, St. Louis, MO, USA), 0.01 % H_2_O_2_ in develop solution (100 mM sodium acetate and 100 mM citric acid). Color development was stopped with 0.8 M H_2_SO_4_ and the reaction was measured at 450 nm. 

For analyzing the antigenicity of purified E2 proteins, we used Strep-Tactin coated plates (IBA Lifesciences, Göttingen, Germany) to capture the StrepII-tagged E2 proteins (1.0 µg/mL in TBS). Subsequent steps were performed as described for the GNL coated plates. 

To analyze the antibodies present in the serum of immunized animals, mice sera was obtained by centrifugation of blood samples at 3600 rpm for 20 min at 4 ℃. ELISA assays were performed as previously described, using total IgG, IgG1, IgG2c and IgG3 antibodies (Santa Cruz Biotechnology, Dallas, TX, USA) [25]. The 96-well plates (NUNC Maxisorp) were coated with 2 μg/mL of a commercial E2 protein from genotype 1a (SinoBiological, Beijing, China), E2 aggregates or E2.C8A monomers diluted in PBS (Invitrogen, Carlsbad, CA, USA) and incubated at 4 ℃ overnight. Next day, plates were washed with PBS-0.01% Tween20 and blocked with 5% skimmed milk in PBS for 2 h at room temperature. Then, plates were washed twice and mice sera diluted in blocking solution were added at different concentrations for 1.5 h at room temperature. Next, plates were washed twice and the appropriate IgG antibody conjugated with HRP and prepared in blocking solution was added for 1 h at room temperature. Finally, after another washing step, the plates were developed as described above.

### 2.6. Determination of Cross-Reactivity against HCV Genotypes

To analyze sera’s cross-reactivity, HEK-293T cells were transfected with pcDNA plasmids expressing the leader peptide of Core and full-length E1 and E2 proteins from different HCV genotypes (1a, 2a, 3a, 4a, 5, and 6) [47]. At 36 h post transfection HEK-293T cells were harvested and lysed with RBS buffer (Tris-HCl 10 mM pH 7.8, NaCl 10 mM and MgCl_2_ 1.5 mM) (60 × 10^6^ cells/mL) for 15 min at 4 ℃. Then, 5% NP-40 (Merck, Darmstadt, Germany) was added followed by 15 min incubation. Cellular extracts were centrifuged at 2000 rpm for 10 min at 4 ℃ and supernatant was collected. Total protein concentration was estimated with BCA kit (ThermoFisher Scientific, Waltham, MA, USA) following manufacturer’s recommendations. ELISA plates were coated with 50 µg of total protein from HEK-293T cellular extracts expressing E1 and E2 proteins from different HCV genotypes and they were reacted with immunized mice sera diluted 1/50.

### 2.7. Sodium Dodecyl Sulfate-Polyacrylamide Gel Electrophoresis (SDS-PAGE)

Purified E2 proteins were analyzed in reducing and non-reducing conditions using Sodium Dodecyl Sulfate-Polyacrylamide Gel Electrophoresis (SDS-PAGE) followed by Coomassie Staining. Briefly, the samples were mixed with loading dye (25 mM Tris, 192 mM Glycine, 20% glycerol, 4% SDS, 0.1% bromophenol blue in milli-Q water) and Dithiothreitol (DTT) (for reducing conditions) and heated at 99 °C for 5 min prior loading on a 4–12% Tris-Glycine gel (Invitrogen, Carlsbad, CA, USA). The gels were run for 30 min at 200 V in running buffer (50 mM MOPS, 50 mM Tris, pH 7.7) at 4 °C. Then, gels were directly stained with Coomassie blue dye (NOVEX^®^, Invitrogen, Carlsbad, CA, USA) and washed with milli-Q water until the band pattern was visible.

### 2.8. Blue Native-PAGE (BN-PAGE)

E2 proteins were analyzed in Blue Native-PAGE (BN-PAGE) followed by Western Blotting as previously described [48,49]. Briefly, purified protein or cells’ supernatant were mixed with loading dye (500 μL20X MOPS Running Buffer (1 M MOPS + 1 M Tris, pH 7.7) + 1 mL 100% Glycerol (Invitrogen) + 50 μL5% Coomassie Brilliant Blue G-250 + 600 μL milli-Q water) and run onto a 4–12% Bis-Tris NuPage (Invitrogen, Carlsbad, CA, USA) in Anode and Cathode buffer (Invitrogen, Carlsbad, CA, USA) at 200 V for 20 min at 4 ℃. For unpurified samples, gels were transferred to a polyvinylidene difluoride (PVDF) membrane, blocked with 5% non-fat skim milk and incubated with 0.25 µg/mL THE^TM^ NWSHPQFEK Tag Antibody (Genscript, Leiden, the Netherlands), followed by extensive washing. The proteins were detected using a HRP-labeled goat anti-mouse antibody (Jackson ImmunoResearch, West Grove, PA, USA). For purified protein samples, BN-PAGE gels were directly stained using the Colloidal Blue Staining Kit (Life Technologies, Carlsbad, CA, USA).

### 2.9. Dynamic Light Scattering (DLS)

Dynamic Light Scattering (DLS) measurements were performed at 20 °C using a Dynapro Nanostar instrument (Wyatt Technology, Santa Barbara, CA, USA), with 10 acquisitions of 5 seconds each. Each protein fraction was centrifuged at 10,000× *g* rpm for 10 min prior to the DLS measurements to remove any trace of aggregates or dust from the sample and 2.5 µg of each fraction was measured. The hydrodynamic radii (Rh) were calculated using Dynamics Analysis software (Wyatt Technology, Santa Barbara, CA, USA), assuming a spherical model.

### 2.10. Mouse Immunizations and Procedures

The animal procedures were performed according to international guidelines, the Ethical Committee of Animal Experimentation of National Center for Biotechnology and the Spanish law under the Royal Decree (RD 53/2013) (permit number PROEX 331/14) and were performed in female C57BL/6JOlaHsd mice 6–8 weeks old, obtained from Envigo. Animals (*n* = 8 per group) were maintained in a pathogen-free animal facility, following the recommendations of the Federation of European Laboratory Animal Science Associations. Priming immunizations were performed at day 0 with protein or virus. The protein inoculums contained 10 μg of each E2 protein adjuvanted with 10 μg of ODN 1826 (Invivogen, San Diego, CA, USA), 2% Alhydrogel (Invivogen, San Diego, CA, USA) and equal parts of Sigma adjuvant Oil (Sigma-Aldrich, St. Louis, MO, USA) in 50 μL of PBS, and were administered intramuscularly in both legs. Viral inoculums contained 1 × 10^7^ plaque forming units (PFU)/mL of MVA-HCV in 200 μL of PBS and were administered intraperitoneally. The animals in the PBS control group received the same adjuvant cocktail as the protein recipient, but no protein. Booster immunizations were performed 15 days post-prime, and were performed as the priming immunizations. At day 10 (adaptive phase) post-boost, half of the mice (*n* = 4) were sacrificed using carbon dioxide (CO_2_), and sera and spleens were collected to analyze the levels of HCV-specific humoral and cellular immune responses, respectively. The other half of mice (*n* = 4) that were left alive for memory immune response study were also bled at day 10 post boost to obtain small serum samples for humoral immune response analysis. Finally, at day 53 (memory phase) the remaining animals were sacrificed and sera and spleens were obtained to analyze the HCV-specific humoral and cellular immune responses, respectively, similarly as for the adaptive phase.

### 2.11. Peptides

HCV peptide pools of the HCV virus, genotype 1a, H77 strain were obtained through Biodefense and Emerging Infectious Research Resources Repository (BEI Resources; National Institute of Allergy and Infectious Disease, National Institutes of Health), and were previously described [24,25]. These peptides cover the entire HCV H77 genome as consecutive 13- to 19-mers overlapping by 11 or 12 amino acids and were used in a final concentration of 1 μg/mL and grouped into 8 pools: core pool (28 peptides), E1 pool (29 peptides), E2 pool (56 peptides), p7 pool (8 peptides), NS2 pool (32 peptides), NS3 pool (98 peptides), NS4 pool comprising NS4A (7 peptides) plus NS4B (40 peptides), and NS5 pool comprising NS5A (71 peptides) plus NS5B (91 peptides). Peptides were used for ex vivo stimulation of splenocytes. 

### 2.12. Intracellular Cytokine Staining (ICS)

We analyzed the magnitude, breadth, polyfunctionality, and memory phenotype of the adaptive and memory HCV-specific CD4^+^ and CD8^+^ T cell immune responses by Intracellular Cytokine Staining (ICS), as previously described [24,25]. Briefly, spleens were collected from immunized mice and processed to obtain splenocytes. Then, splenocytes were stimulated for 6 h at 37 °C with 1 μg/mL of different HCV peptide pools of the HCV genotype 1a, H77 strain and stained with the appropriate fluorochrome-conjugated antibodies against different surface markers: CD4-APC-Cy7, CD8-V500, CD62L-Alexa700, CD127-PerCP-Cy5.5, and CD107a-FITC (all from BD Biosciences, Franklin Lakes, NJ, USA). Subsequently, cells were fixed and permeabilized with Cytofix/Cytoperm kit (BD Biosciences, Franklin Lakes, NJ, USA) and the intracellular cytokines were stained using the appropriate fluorochrome-conjugated antibodies: IFN-γ-PECy7 (BD Biosciences, Franklin Lakes, NJ, USA), TNF-α-PE (eBioscience, San Diego, CA, USA) and IL-2-APC (BD Biosciences, Franklin Lakes, NJ, USA). Cells were passed through a Gallios flow cytometer (Beckman Coulter, Brea, CA, USA) and data analysis was carried out using the FlowJo (version 8.8.7) program.

### 2.13. Statistical Analysis

Statistical analysis of the ICS data was done as previously described [50,51]. Statistical significance in the ELISA assays was determined using one-way ANOVA with logarithmic transformation of data. Statistical significance in the ELISA assays comparing adaptive versus memory immune response was determined using the Homl-Sidak method (T test), with alpha = 5%. Statistically significant differences were denoted as follows: * *p* < 0.05; ** *p* < 0.005; *** *p* < 0.001.

## 3. Results

### 3.1. Design and Generation of a Recombinant HCV E2 Protein Lacking Eight Cysteine Residues

We generated two E2 variants to evaluate the impact of the 8 cysteine residues substitutions in their in vitro properties and in vivo immunogenicity. Firstly, we generated E2_MPER_ protein, which encompasses the complete ectodomain of E2 (amino acids 384–715, H77 numbering [41]), including the flexible stem region (amino acids 675–698) [52] and the hydrophobic MPER (amino acids 698–715). We hypothesized that the hydrophobicity of the MPER might contribute to the formation of unwanted aggregates, analogous to what has been observed for soluble HIV-1 glycoproteins [53]. Therefore, we also generated a soluble E2 protein lacking the MPER by truncating E2 at Asp698 (termed E2). 

**Figure 1:**
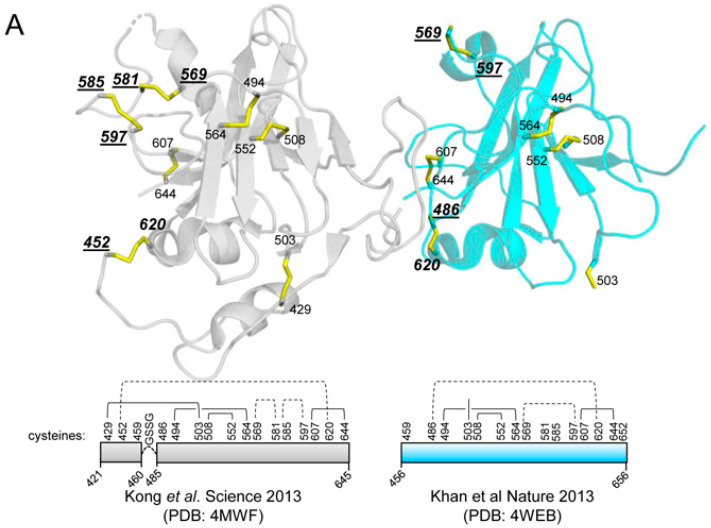

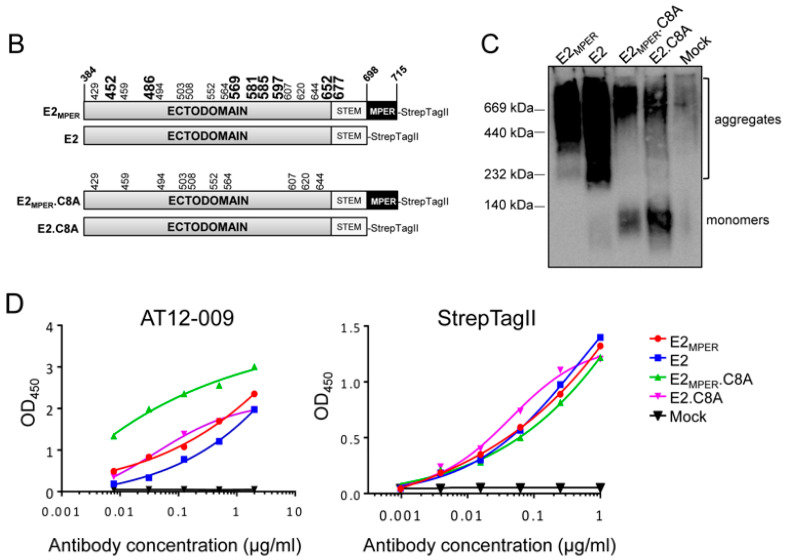
Design and characterization of recombinant E2 proteins. (**A**) Overview of the disulfide bond networks in the first two E2 core crystal structures [37,38]. Top: the cysteines in disulfide bonds (highlighted in yellow sticks) that were not ubiquitously assigned are indicated in italic bold. The cysteines changed to alanine in E2_MPER_.C8A and E2.C8A are underlined (see B). *Bottom:* scheme of the disulfide bond networks of both structures with the non-ubiquitous disulfides depicted with dotted lines. (**B**) Scheme of the different recombinant E2 proteins generated. Ectodomain (residues 384–674) is followed by a stem region (residues 675–698). The membrane-proximal external region (MPER) (residues 699–715) was included in E2_MPER_ and in E2_MPER_.C8A. All proteins contain a Strep-tagII sequence in the C-terminal region. The aminoacid positions (H77 numbering) of the cysteines present in E2 are shown in vertical numbers, and those in bold were substituted by alanines in E2.C8A proteins. (**C**) Blue Native-PAGE (BN-PAGE) analysis of supernatants from HEK-293T cells transfected with the different E2 constructs followed by Western Blotting using an anti-Strep-tagII antibody. (**D**) Lectin-capture ELISA with unpurified supernatants from transfected HEK-293T cells using AT12-009 (an anti-E2 antibody) and anti-Strep-tagII antibody (used to check the expression). The results for AT12-009 are shown with normalized data using anti-Strep-tagII at 0.6 µg/mL.

Most recombinant soluble E2 glycoproteins produce high amounts of disulfide-linked aggregates, while E2 core proteins that lack the three variable regions, the stem and MPER mostly produce monomers [37,38,54]. Thus, we aimed to design an E2 immunogen that preserved the variable regions and the stem, but with a higher tendency to form monomers by strategically removing cysteine residues that cause undesired disulfide-linked aggregates. Interestingly, the first two crystal structures of the E2 core revealed different disulfide bond networks and only six out of eighteen cysteines are located in disulfide bonds that were resolved in both structures (C494–C564; C508–C552; and C607–C644) [36,37,38,39] (Figure 1A). This suggested that some of the other disulfide bonds might be redundant for the folding of antigenically correct E2 core. We hypothesized that the discordant disulfide bonds might contribute to aggregate formation in the context of the complete E2 ectodomain. Therefore, we mutated the eight cysteines that have not ubiquitously been assigned to a specific disulfide bond in the first two published E2 structures and those located in the stem [37,38] (cysteines at positions 452, 486, 569, 581, 585, 597, 652, and 677) to alanines, generating the E2.C8A and E2_MPER_.C8A proteins. Of note, omission of these cysteines abrogated HCV infectivity, but did not affect binding of E2 to CD81 in an earlier study [54]. A Strep-tagII was added to the C-terminus of each construct to facilitate purification. A scheme depicting the different E2 variants (E2, E2_MPER_, E2.C8A and E2_MPER_.C8A) is shown in Figure 1B. 

### 3.2. E2 Proteins Lacking Eight Cysteines are Expressed Less as Aggregates 

The four E2 constructs were transfected in HEK-293T cells and the supernatants were analyzed 48 h post-transfection. BN-PAGE followed by Western Blotting showed that all the E2 proteins were secreted into the extracellular media (Figure 1C and Figure S1). Importantly, the E2 and E2_MPER_ proteins, containing all cysteines, seemed to almost exclusively form higher molecular weight aggregates, while the E2.C8A and E2_MPER_.C8A cysteine mutants are expressed with lower levels of aggregates and mostly formed lower molecular weight monomers. This suggests that the removal of the cysteines did not affect E2 production, but strongly decreased E2 aggregation (Figure 1C and Figure S1). Furthermore, the removal of the MPER increased the amount of monomers that were detected in Western Blot (Figure 1C and Figure S1).

Next, we analyzed the binding of the secreted E2 proteins to an anti-E2 antibody. Thus, the E2-containing supernatants were probed by ELISA with AT12-009, an anti-CD81 binding site bNAb [6], and an anti-Strep-tagII antibody used as an expression control. The expression was very similar between the constructs, but supernatants containing E2 proteins lacking eight cysteines (E2_MPER_.C8A and E2.C8A) showed better binding to AT12-009 than the wild type E2 proteins (E2_MPER_ and E2) (Figure 1D). We chose to continue with E2 and E2.C8A for further analysis, because the variants with MPER contain more unwanted aggregates.

### 3.3. E2.C8A Is Expressed Mainly as Monomers

Next, both E2 and E2.C8A proteins were produced and purified with the aim of obtaining high quality proteins that could be further characterized in vitro and subsequently used for immunization experiments in mice to study their immunogenic properties in vivo. 

**Figure 2:**
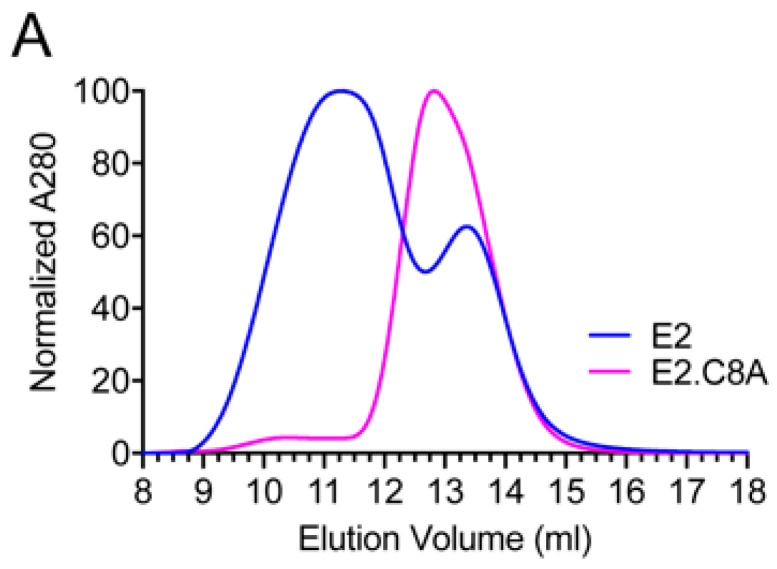

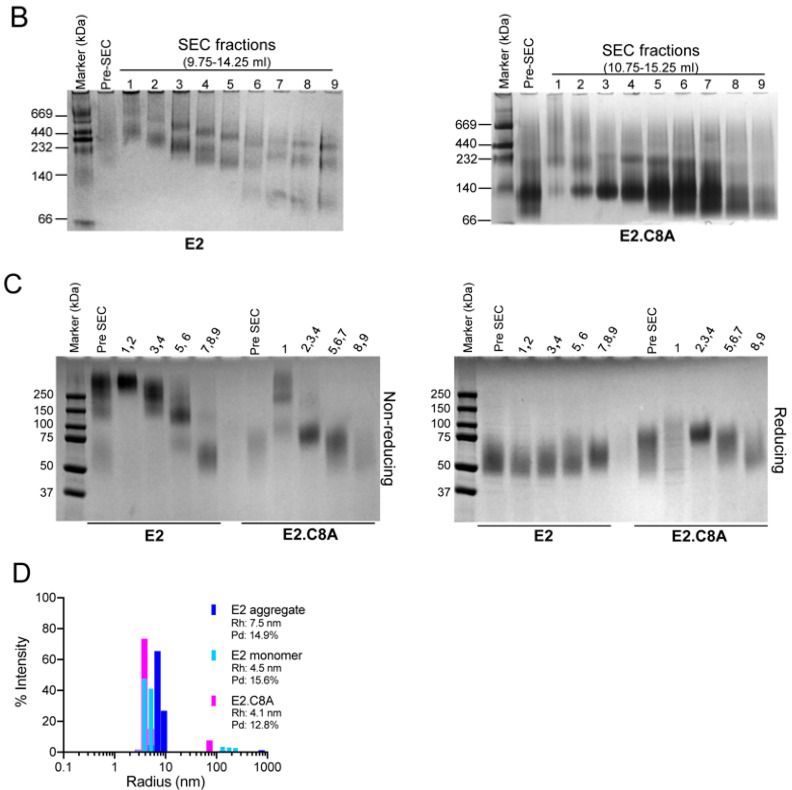
Characterization of purified E2 and E2.C8A. (**A**) Size-exclusion chromatography (SEC) profiles of StrepTactin-purified E2 and E2.C8A proteins. (**B**) BN-PAGE gels loaded with 5 μg of proteins from each fraction followed by Coomassie blue staining of the SEC fractions obtained in *A*. Numbers indicate the fractions in 0.5 mL increments. Pre-SEC corresponds to the StrepTactin-purified protein sample before SEC. (**C**) Sodium Dodecyl Sulfate-Polyacrylamide Gel Electrophoresis (SDS-PAGE) analysis followed by Coomassie blue staining under non-reducing (left panel) and reducing (right panel) conditions of pooled fractions, corresponding to the same fractions as in Figure 2B. (**D**) Dynamic Light Scattering (DLS) profiles of E2 aggregates (E2 fractions 1–2, see B left panel), E2 monomers (E2 fractions 7–9, see B left panel) and E2.C8A monomers (E2.C8A fractions 8–9, see B right panel). Rh: hydrodynamic radius; Pd: polydispersity.

The E2 and E2.C8A constructs were transfected in HEK-293F cells and six days after transfection their supernatants were passed over a Strep-Tactin^®^ XT column. Average yields (*n* = 2) were ~8.5 (±0.4) mg/L for E2 and ~3.9 (±1.5) mg/L for E2.C8A. The size-exclusion profile of purified E2 was relatively broad and consisted of two peaks (peaks around 11.5 and 13.2 mL), while the profile of E2.C8A was much narrower and showed one large peak around 12.8 mL (Figure 2A). 

BN-PAGE of the different fractions confirmed that E2 mostly formed high molecular weight aggregates (Figure 2B, left panel), while E2.C8A consisted mostly of low molecular weight proteins that could represent the protein in monomeric form (Figure 2B, right panel). Next, we pooled the fractions of similar molecular weight and performed SDS-PAGE under reducing and non-reducing conditions to characterize the effect of the cysteine removal in E2.C8A (Figure 2C). We found that the higher molecular weight fractions of E2 (molecular weight of more than 140 kDa) (Figure 2B, fractions 1–6) and E2.C8A (Figure 2B, fraction 1) also formed high molecular weight variants under non-reducing conditions (Figure 2C, left panel), demonstrating that the omitted cysteines are the main cause of inter-E2 disulfide bond formation. The lower molecular weight variants (less than 140 kDa) of E2 (Figure 2B, fractions 7–9) and E2.C8A (Figure 2B, fractions 2–9) showed distinct lower molecular weight bands under non-reducing conditions (Figure 2C, left panel). Interestingly, under reducing conditions, while E2 showed bands of similar molecular weight in all fractions (between 50–60 kDa), E2.C8A showed different molecular weight sizes among its fractions (ranging from 45–75 kDa) (Figure 2C, right panel). The data suggest that E2 and E2.C8A have differences in glycosylation [55]. Unedited and non-cropped Coomassie gels from Figure 2A,B can be found in Supplementary Materials Figure S2.

The differences in size were also confirmed by DLS, where E2 aggregates (fractions 1 and 2 combined) had a hydrodynamic radius (R_h_) of 7.5 nm, whereas the monomeric fractions of E2 and E2.C8A were similar in size (4.5 and 4.1 nm, respectively) (Figure 2D). All samples were relatively monodisperse with a polydispersity of <15% [56].

### 3.4. E2.C8A Monomers Are Efficiently Recognized by Broadly Neutralizing Antibodies (bNAbs) and CD81

**Figure 3:**
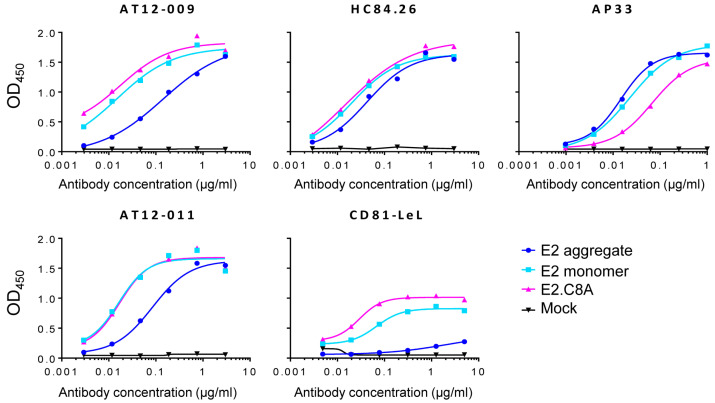
Antigenicity of E2 and E2.C8A. Strep-Tactin coated ELISA plates were loaded with 1.0 μg/mL of StrepTactin/SEC-purified E2 (aggregates and monomers) or E2.C8A (monomer) proteins and were reacted against serial dilutions of different antibodies.

An important component in the design of recombinant vaccine proteins is to define their antigenic properties. To this end, several monoclonal bNAbs targeting different E2 epitopes were tested for binding to E2 aggregates, E2 monomers and E2.C8A monomers. AP33 is a bNAb that recognizes a linear epitope [57], while AT12-009 and AT12-011 recognize conformational epitopes in domains B and C, respectively. Both antibodies recognize E2 of genotypes 1–6, but only AT12-009 also neutralizes viruses of all genotypes, whereas AT12-011 only neutralizes viruses of genotypes 1a, 1b and 2a [6]. HC84.26 is one of the members of the family of antibodies that bind to a conformational epitope in antigenic domain D and neutralizes viruses from most genotypes [58,59]. Thus, ELISAs were carried out to determine if the previously purified E2 aggregates (fractions 1–2 in Figure 2C), E2 monomers (fractions 7–9 in Figure 2C) and E2.C8A (fractions 8–9 in Figure 2C) are recognized by bNAbs. Conformational antibodies AT12-009, AT12-011, and HC84.26 recognized the E2.C8A and E2 monomers more efficiently than E2 aggregates. However, in the case of the AP33 linear antibody, E2 aggregates and E2 monomers were recognized more efficiently than E2.C8A (Figure 3).

Finally, to characterize the binding of the different E2 proteins to the CD81 receptor (one of the main HCV receptors on hepatocytes), ELISAs were performed using a fusion construct consisting of the mouse antibody Fc-tail fused to the large extracellular loop (LEL) of the CD81 receptor (CD81-LEL), which presents the specific binding site of the E2 protein [60,61]. Again, E2.C8A and E2 monomers reacted more strongly with CD81-LeL than aggregated E2 (Figure 3). 

### 3.5. E2 Aggregates and E2.C8A Monomers Induced Adaptive HCV-Specific CD4^+^ and CD8^+^ T Cell Immune Responses in Immunized Mice When Combined with MVA-HCV in Heterologous Prime/Boost Regimens

**Figure 4:**
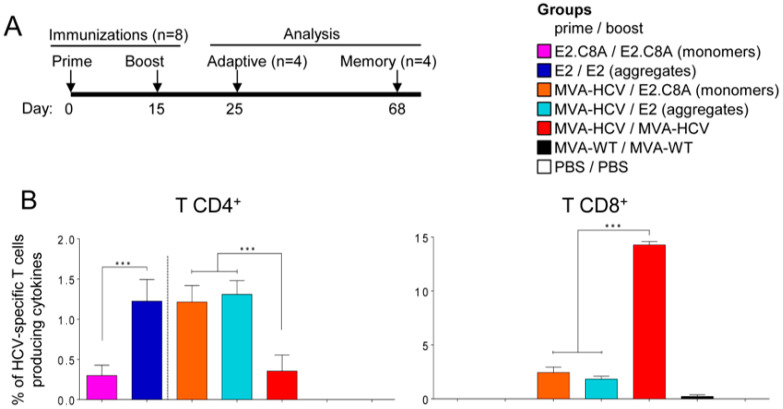

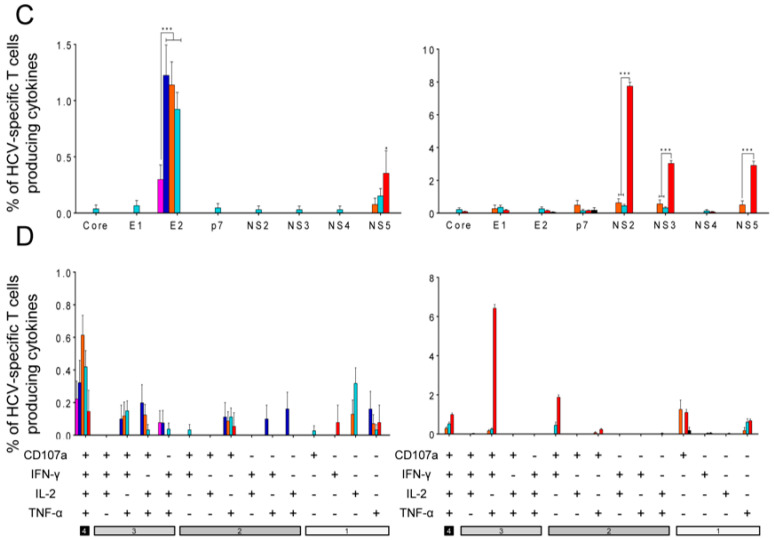
Adaptive CD4^+^ and CD8^+^ HCV-specific T cell immune responses in immunized mice. (**A**) Mouse immunization schedule. Eight C57BL/6 mice were immunized with E2 proteins, MVA-HCV, MVA-WT, or PBS at days 0 and 15. At day 25, serum was taken from each mouse for analyzing humoral responses (see Figures 7 and 8), after which half of the mice were sacrificed to analyze T cellular immune responses in the adaptive phase. At day 68, the remaining four mice were sacrificed for analyzing responses in the memory phase. (**B**) Magnitude of the total HCV-specific CD4^+^ and CD8^+^ T cell responses after stimulation of splenocytes with the different HCV peptide pools. The total value in each group represents the sum of the percentages of CD4^+^ and CD8^+^ T cells secreting CD107a and/or IFN-γ and/or IL-2 and/or TNF-α against HCV antigens. Dashed line separates groups stimulated only with E2 peptide pools (left) from groups stimulated against all HCV antigens (right). (**C**) Breadth of the HCV-specific T cell response shown as the percentage of cells secreting CD107a and/or IFN-γ and/or IL-2 and/or TNF-α against each HCV peptide pool. (**D**) Polyfunctionality of the HCV-specific T cell response shown as the combined production of CD107a and/or IFN-γ and/or IL-2 and/or TNF-α against all HCV peptide pools. *p* values indicate significant response differences between immunization groups (*** *p* < 0.001).

It has been recently reported that aggregated E2 is more immunogenic than E2 monomers [62]. Thus, to study the immunogenicity of the different oligomeric forms of E2, we selected the aggregate fractions of E2 (fractions 1–2, Figure 2C) and the monomeric fractions of E2.C8A (fractions 8–9, Figure 2C). Groups of mice were immunized with protein in homologous prime/boost protocols (E2/E2 or E2.C8A/E2.C8A) by intramuscular route or in heterologous prime/boost protocols consisting of a prime with MVA-HCV and a protein boost with E2 or E2.C8A (Figure 4A). The adaptive HCV-specific CD4^+^ and CD8^+^ T cell responses were determined by ICS 10 days after the boost in HCV peptide stimulated-splenocytes.

The results in the adaptive phase showed that the total immune response of HCV-specific CD4^+^ T cells was similar for E2/E2, MVA-HCV/E2.C8A, and MVA-HCV/E2, while the groups E2.C8A/E2.C8A and MVA-HCV/MVA-HCV generated the lowest magnitude of CD4^+^ T cells (Figure 4B, left panel). It should be noted that the responses measured for MVA-HCV primed or prime/boosted mice are the sum of the responses to all HCV proteins (from core to NS5), while the same measurements only represent the E2-directed responses in the case of protein-only immunized groups (E2/E2 or E2.C8A/E2.C8A). This distinction is represented by a discontinuous line in Figure 4B in left panel.

When dissecting the total T CD4^+^ response by target HCV protein, we observed that the majority of the HCV-specific responses are directed to E2, with no differences between E2/E2, MVA-HCV/E2, and MVA-HCV/E2.C8A. Only the MVA-HCV/MVA-HCV group mainly induced responses to NS5 and not to E2 (Figure 4C, left panel).

The quality of the immune response of the CD4^+^ T cells generated by the different immunization groups was analyzed by measuring the pattern of cytokine production (IFN-γ, TNF-α and/or IL-2) plus its cytotoxic potential (CD107a as marker of degranulation). In general, the response generated by all the immunization groups was highly polyfunctional, with majority of cells producing all four cytokines (Figure 4D, left panel).

The MVA-HCV/MVA-HCV group elicited the most potent HCV-specific CD8^+^ T cell responses in the adaptive phase (Figure 4B, right panel), which were mainly directed against NS2, NS3, and NS5 (Figure 4C, right panel). The groups immunized with only E2 or E2.C8A proteins in the homologous regimens did not elicit detectable CD8^+^ T cell responses, while the combined MVA-HCV/E2 or MVA-HCV/E2.C8A groups elicited detectable CD8^+^ T cell responses (Figure 4B, right panel) that were mainly directed against NS2 and NS3, and were significantly lower than those induced by MVA-HCV/MVA-HCV (Figure 4C, right panel). Most HCV-specific CD8^+^ T cells were polyfunctional, as demonstrated by their simultaneous expression of CD107a, IFN-γ and TNF-α or CD107a, IFN-γ, TNF-α and IL-2 (Figure 4D, right panel). 

### 3.6. MVA-HCV Priming Before an E2 Protein Boost Increases the Memory CD4^+^ T Cell Responses 

**Figure 5:**
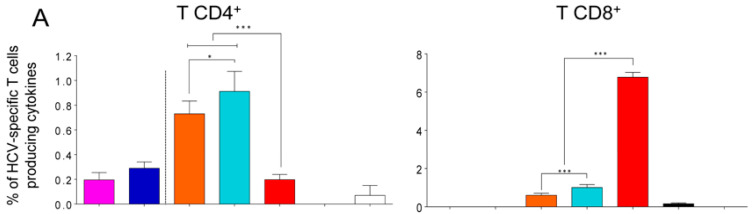

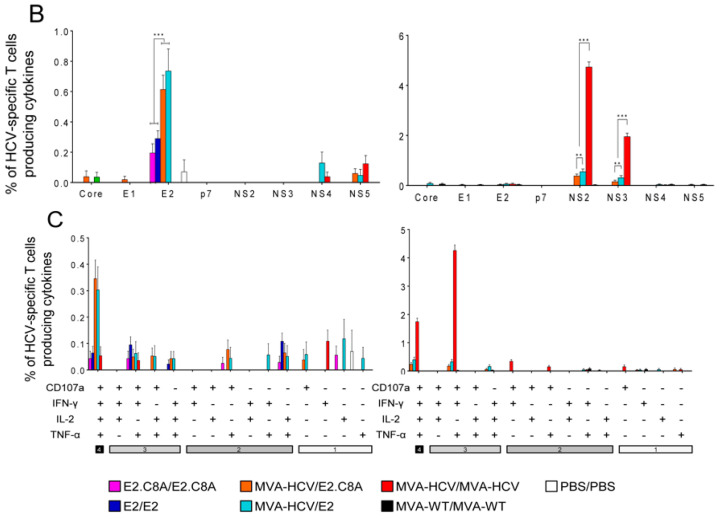
Memory CD4^+^ and CD8^+^ HCV-specific T cell immune responses in immunized mice. *p* values indicate significant response differences between immunization groups (* *p* < 0.05; ** *p* < 0.005; *** *p* < 0.001). (**A**) Magnitude of the total HCV-specific CD4^+^ and CD8^+^ T cell responses after stimulation of splenocytes with the different HCV peptide pools. The total value in each group represents the sum of the percentages of CD4^+^ and CD8^+^ T cells secreting CD107a and/or IFN-γ and/or IL-2 and/or TNF-α against HCV antigens. Dashed line separates groups stimulated only with E2 peptide pools (left) from groups stimulated against all HCV antigens (right). (**B**) Breadth of the HCV-specific T cell response shown as the percentage of cells secreting CD107a and/or IFN-γ and/or IL-2 and/or TNF-α against each HCV peptide pool. (**C**) Polyfunctionality of the HCV-specific T cell response shown as the combined production of CD107a and/or IFN-γ and/or IL-2 and/or TNF-α against all HCV peptide pools.

To study the cellular immune response at the memory phase, half of the animals in each group were sacrificed 53 days after the boost (i.e., day 68 in Figure 4A). The MVA-HCV/E2 and MVA-HCV/E2.C8A groups induced the highest CD4^+^ T cell responses (Figure 5A, left panel) and were still mostly directed to E2 (Figure 5B, left panel). Importantly, the E2-specific CD4^+^ T cell responses were significantly higher in the MVA-HCV/E2 and MVA-HCV/E2.C8A groups compared to the E2/E2 or E2.C8A/E2.C8A protein-only groups, suggesting that the MVA-HCV prime helped to prolong the memory CD4^+^ T cell response. The responses of the MVA-HCV/MVA-HCV group were mostly directed to NS5 and no E2 responses were detected in the memory phase (Figure 5B, left panel). All HCV-specific CD4^+^ T cells were polyfunctional, with the majority of them being quadruple producers (CD107a+IFN-γ+TNF-α+IL-2) (Figure 5C, left panel). 

Only the MVA-HCV/MVA-HCV immunized animals elicited potent memory CD8^+^ T cell responses (Figure 5A, right panel), which were directed against NS2 and NS3 (Figure 5B, right panel), and the HCV-specific CD8^+^ T cells were mainly triple (CD107a+IFN-γ+TNF-α) and quadruple (CD107a+IFN-γ+TNFα+IL-2) producers (Figure 5C, right panel). Weak, but detectable, memory CD8^+^ T cell responses against NS2 and NS3 were induced by both MVA-HCV/E2 and MVA-HCV/E2.C8A groups (Figure 5A,B, right panel), which were also polyfunctional (Figure 5C, right panel). 

**Figure 6:**
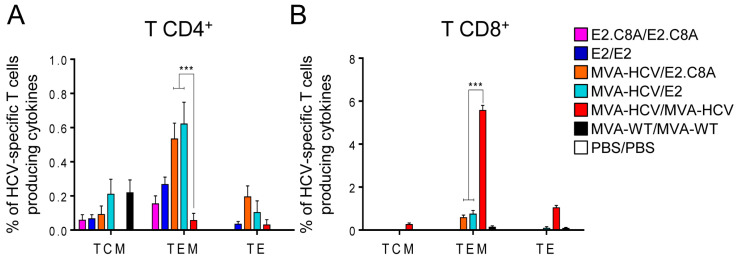
Memory phenotype of T cell immune responses in immunized mice. Phenotypic profiles of splenocytes from the memory phase (*n* = 4 mice per group) were analyzed by Intracellular Cytokine Staining (ICS) assay. Graphs indicate the percentages of CD4^+^ (left) and CD8^+^ T (right) central memory (TCM; CD127^+^ CD62L^+^), effector memory (TEM; CD127^+^ CD62L^−^), and effector (TE; CD127^−^ CD62L^−^) cells expressing CD107a and/or producing IFN-γ and/or TNF-α and/or IL-2 against all genotype 1a HCV peptide pools. *p* values indicate significant response differences between immunization groups (*** *p* < 0.001).

To study the memory phenotype of the HCV-specific CD4^+^ T cells, we analyzed the expression of CD127 and CD62L surface markers on CD4^+^ and CD8^+^ HCV-specific T cells at 53 days after the boost. For CD4^+^ T cells, the majority of the responses were mainly of a T effector memory phenotype (TEM) with no major differences between the groups (Figure 6, left panel). The HCV-specific memory CD8^+^ T cells were mainly of a TEM phenotype with no major differences between the groups (Figure 6, right panel).

### 3.7. E2 Aggregates Induced Greater Humoral Immune Response than E2.C8A Either Alone or in Combination with MVA-HCV 

**Figure 7:**
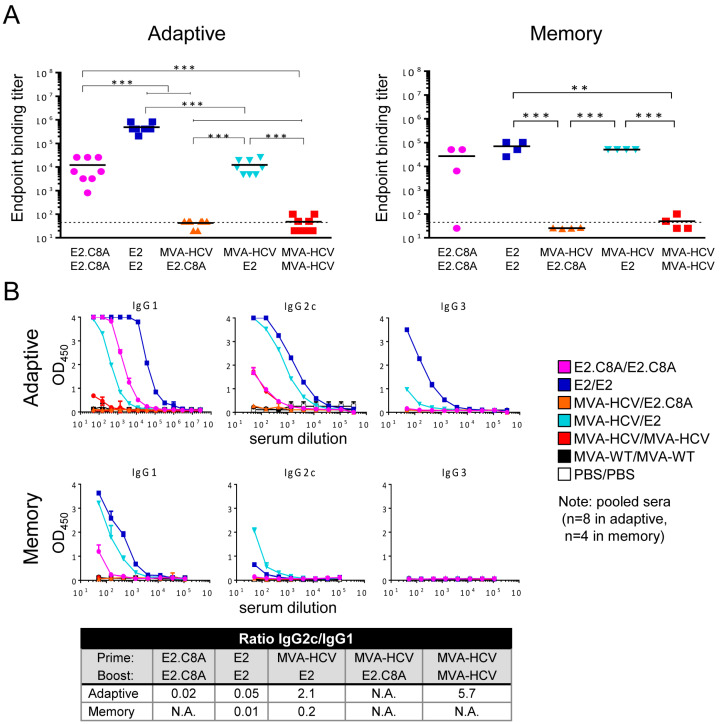
HCV-specific humoral immune responses elicited in immunized mice. (**A**) Serum IgG binding titers against commercially available H77 E2 protein were determined by ELISA 10 days (left) and 53 days (right) post-boost in individual mice. The endpoint titer is the serum dilution at which the absorbance is three times higher than the absorbance value of the control group (MVA-WT/MVA-WT). *p* values indicate significant response differences between immunization groups (** *p* < 0.005; *** *p* < 0.001). (**B**) Detection of IgG1, IgG2c, and IgG3 levels against H77 E2 in pooled serum from immunized mice at 10 days (upper graphs) and 53 days (lower graphs) post-boost. The table shows the IgG2c/IgG1 ratio of the different groups at adaptive and memory phases. N.A. = not available.

We analyzed the humoral immune responses induced by the different immunization groups against a commercially available H77 E2 protein (see Methods) using ELISA. In the adaptive phase, the E2/E2 aggregate group generated higher levels of total IgG antibodies (geometric mean binding titer: ~500,000) compared to the E2.C8A/E2.C8A monomer group (geometric mean titer: ~12,000) (Figure 7A, left panel). In addition, the E2 aggregates induced a significantly stronger antibody response than the E2.C8A monomer as boosts for the MVA-HCV primed immunizations, at least as measured against the commercially available E2 probe (titers: ~12,000 and ~40, respectively). The MVA-HCV/MVA-HCV immunization group induced very low titers of antibodies. Significantly, during the memory phase the antibody titers remained high in the homologous E2/E2 and in the heterologous MVA-HCV/E2 groups (titers: ~70,400 and ~51,200, respectively) (Figure 7A, right panel). Next, we compared the antibody levels elicited by the different groups in adaptive phase versus memory phase in order to study the decay of antibody levels. Surprisingly, the MVA-HCV/E2 group showed a slightly increased of antibody titer in the memory phase, while the rest of the groups showed no change or lower antibody titers in memory phase compared to the adaptive phase (Figure 7A).

The study of IgG isotypes is an indirect measure of cell polarization towards a Th1 or Th2 response, so that if the levels of IgG2 are higher than those of IgG1, the response is mainly skewed towards Th1 cells [63]. Therefore, to elucidate the polarization of the CD4^+^ T cell response towards Th1 or Th2, we pooled mouse serum from different groups and performed ELISA to determine the levels of IgG1, IgG2c, and IgG3 antibody isotypes in the adaptive and memory phases. In the adaptive phase, the E2/E2 and E2.C8A/E2.C8A immunized mice induced antibodies that were mostly of the IgG1 isotype, suggesting that the CD4^+^ T cell responses were skewed to Th2 (Figure 7B, upper panel and table). IgG1 levels remained high for these groups in the memory phase (Figure 7B, lower panel). However, the MVA-HCV/E2 immunized mice also induced relatively high levels of IgG2c isotype, which were still detectable in the memory phase (Figure 7B, lower panel), showing an IgG2c/IgG1 ratio of 2.1, indicating a predominant Th1 response in the adaptive phase. 

**Figure 8:**
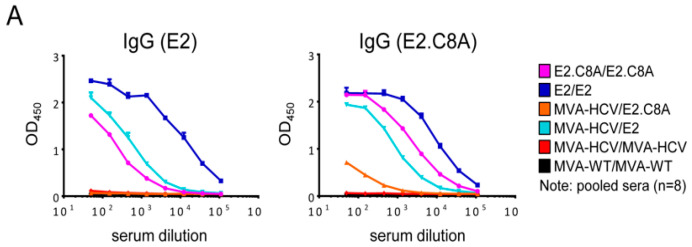

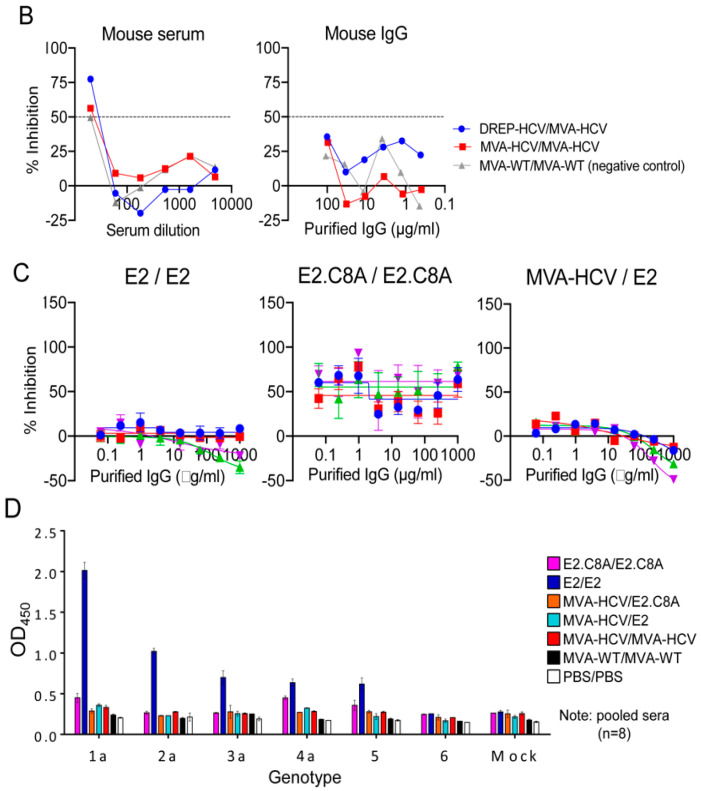
Binding reactivity and neutralization activity of the mice sera. (**A**) Total IgG binding levels against E2 aggregates (left panel) or E2.C8A monomers (right panels) in pooled serum from immunized mice at 10 days post-boost. (**B**) A pilot neutralization experiment comparing the inhibitory activity of serially diluted mice sera obtained from a previous immunization with DREP-HCV/MVA-HCV, MVA-HCV/MVA-HCV, and MVA-WT/MVA-WT [25] and the corresponding purified IgG samples against H77 HCV pseudo particles (HCVpp). (**C**) Neutralization assay curves showing inhibition of H77 HCVpp infectivity by serially diluted purified IgG of four mice sera from immunized mice with E2/E2, E2.C8A/E2.C8A and MVA-HCV/E2 at 10 days post-boost. (**D**) Binding of pooled sera (at a 1/50 dilution, *n* = 8 mice/group) from the adaptive phase (10 days post-boost) to lysed HEK293T cells expressing core-E1-E2 from different HCV genotypes was determined by ELISA.

Next, we analyzed by ELISA the adaptive humoral immune responses induced by the different immunization groups against E2 aggregates or E2.C8A monomers in pooled mouse serum samples (Figure 8A). The E2/E2 group induced the highest levels of total IgG antibodies against aggregated E2, followed by MVA-HCV/E2 and E2.C8A/E2.C8A (Figure 8A, left panel). E2/E2 also induced the highest binding Ab levels against E2.C8A, but the titer of E2.C8A/E2.C8A against E2.C8A were higher than those elicited by the MVA-HCV/E2 group (Figure 8A, right panel). These results demonstrate that the E2 protein aggregates induced the highest levels of IgG titers irrespective of the tested antigen and it suggests that E2.C8A elicits serum responses that are more specific for E2.C8A monomers. 

Finally, we determined the neutralizing serum activity of these immunized mice. However, mice sera can contain viral inhibitory components that can interfere with neutralization assays as reported for HIV-1 [46]. To test this, we first performed a small pilot HCV pseudoparticle (HCVpp) neutralization assay [64] using three serum samples from mice immunized with DREP-HCV/MVA-HCV, MVA-HCV/MVA-HCV and MVA-WT/MVA-WT [25]. We found that even a negative control serum (MVA-WT/MVA-WT) showed ~50% neutralizing activity at a 1/20 dilution, while the purified IgG samples did not (Figure 8B). Therefore, we decided to perform the HCV pseudoparticle (HCVpp) neutralization assay using purified IgG samples to avoid non-specific serum inhibition. However, we did not detect any neutralizing activity for any of the analyzed groups (E2/E2, E2.C8A/E2.C8A and MVA-HCV/E2) (Figure 8C). The lack of HCV neutralization in mouse serum will be considered under the Discussion section.

### 3.8. E2/E2 Immunized Mice Elicited Cross Reactive Responses Against Other HCV Genotypes 

To elucidate whether antibody responses were also reactive against other HCV genotypes, the pooled mice sera from the adaptive phase were tested against cell extracts containing core-E1-E2 proteins from six different genotypes (1 to 6) in ELISA. The results showed that the antibodies induced by the E2/E2 immunization group, besides binding to proteins from the homologous genotype 1a, also cross-reacted with proteins from genotypes 2–5 (Figure 8D). The other immunization groups did not induce detectable levels of antibodies against proteins from genotypes 2–6. 

## 4. Discussion

To generate an effective HCV vaccine, both optimized immunogens and novel immunization strategies are necessary. Here, we report the design of a non-aggregating well-folded recombinant E2 immunogen and its use in MVA-based prime/boost immunization strategies that are aimed at improving anti-HCV responses. In the present study, we successfully generated a non-aggregating form of recombinant E2 (E2.C8A) by strategically removing cysteine residues. We did remove the MPER from E2, because it did not seem to affect antigenicity and it is known that a similar region in the HIV-1 glycoprotein can induce aggregation [53]. The 18 cysteines in E2 have a multifaceted role, as they are crucial in protein structure, but also in protein rearrangement upon viral attachment, probably taking part in fusion conformational changes. Given their importance in functionality, it is surprising that only three disulfide bridges (C494–C564, C508–C552, and C607–C644) are shared in the first two published crystal structures [37,38]. Later, crystal structures of a more complete E2 confirmed the existence of these three disulfide bonds, but also showed additional disulfide bonds that were not observed before [39]. We found that E2.C8A interacted equally well with monoclonal conformational antibodies as conventional E2 monomers and even better than E2 aggregates, which corresponds to the findings reported by a recent study on another recombinant E2 containing less cysteines [65]. 

These findings surprised us since the omitted cysteines are highly conserved across genotypes and are important for proper E1E2 folding and crucial for viral fitness [54]. Therefore, we hypothesize that several of the cysteines missing in E2.C8A might directly interact with E1 or are crucial during viral entry. In this regard, it is important to highlight that E1E2 might contain reduced cysteines that are crucial for virus entry [66]. Furthermore, we also noticed that the small amount of aggregates in the produced E2.C8A is also disulfide bonded (Figure 1C). This suggests the many cysteines in E2 might induce or enhance aggregation. We hypothesize that this might be due to the lack of interaction with E1 acting as a chaperone for native folding of E2 as presented in the complete E1E2 complex. Similarly, overexpressing of HIV-1 gp120 without gp41 can also cause the formation of aberrant disulfide-linked gp120 [67]. One could speculate that this promiscuous disulfide bridge network of E2 might help to prevent E1E2 of being recognized by the immune system by creating heterogeneity. 

Unexpectedly, the E2.C8A monomers were less immunogenic than E2 aggregates in vivo, despite the fact that the E2.C8A interacted more efficiently with bNAbs in vitro. This might be explained if the aggregates behave as coincidental nanoparticles facilitating multivalent presentation of E2 epitopes. It is well-established that nanoparticle presentation of viral glycoproteins improves (humoral) immune responses [68,69,70]. Several mechanisms explain how nanoparticles and aggregates can enhance the immune response. Most importantly, higher antigen valency improves B cell receptor activation and increases complement system activation [71]. Importantly, our results confirm other studies that reported that a higher oligomeric state of E2, either as aggregate [62,65] or when presented on nanoparticles [72,73,74], enhances the immune response. Furthermore, a recent study showed that aggregated forms of E2, including a variant containing less cysteine residues, induce higher quality Abs in guinea pigs [65]. In summary, optimized forms of E2.C8A and nanoparticle presentation are promising strategies to improve and increase NAb responses. 

During acute primary infection, the appearance of potent, broad, and polyfunctional HCV-specific T cell responses is a hallmark of patients that resolve the infection, leading to long-lived memory T cells. MVA-based vectors are highly immunogenic and trigger potent CD8^+^ T cell responses, but weak antibody responses, while protein immunizations are poor CD8^+^ T cell inducers and induce stronger antibody responses. In the present study, we found that the homologous MVA-HCV only or protein-only immunization regimens induced strong CD8^+^ T cell (MVA-HCV driven) or antibody responses (protein-driven), but not both. In contrast, the combined strategy employing MVA-HCV as a prime followed by E2 protein as a boost, resulted in a more balanced T/B cell response. This response included CD4^+^ and CD8^+^ HCV-specific T cells and higher levels of (cross-reactive) binding antibodies in adaptive and memory phases. Importantly, this CD4^+^ T cell response in heterologous immunization groups was of higher magnitude than in the MVA-HCV/MVA-HCV group, while the CD8^+^ T cell response was higher in the MVA-HCV/MVA-HCV group. Additionally, MVA-HCV/E2 not only elicited both T/B immune responses, but also maintained the antibody levels in the memory phase.

Furthermore, the T cell responses induced by the heterologous regimens were highly polyfunctional (quadruple and triple cytokine producers) and broad, with CD4^+^ T cells mainly directed against E2, and CD8^+^ T cells against NS2 and NS3 HCV proteins. It has been described that CD8^+^ T cells targeting NS3 protein play an important role in resolving infection in chimpanzees [75], which might be related to its potent IFN inhibitory activity [76,77]. Additionally, NS3 is a relatively conserved HCV protein, which makes it an attractive target for T cell vaccines [78]. Furthermore, the MVA-HCV/E2 proteins vaccination regimen elicited HCV-specific memory T cells expressing CD127 (TCM and TEM), which is part of the IL-7 receptor and is predictive for the number of memory T cells generated following vaccination [79]. 

Given the complexity of the immune response against HCV in infected patients, it is not still clear which cytokines and T CD4^+^ responses (Th1 or Th2) favor the control of the disease. Some studies have reported the prevalence of Th1 responses in patients that achieved sustained virological response [80,81], but others documented dominant Th2 type immune response or presence of both Th1/Th2 responses in chronic HCV patients [82,83,84,85,86,87]. In the present study, protein only immunizations generated higher levels of IgG1 than IgG2c, indicating a Th2 type response. However, groups MVA-HCV/E2 and MVA-HCV/MVA-HCV induced a higher IgG2c/IgG1 ratio in the adaptive phase, indicating a Th1 type response.

The analysis of the binding IgG levels directed either against E2 aggregates or E2.C8A monomers showed that the E2/E2 group induced the highest levels of total IgG antibodies against aggregated E2, while E2.C8A/E2.C8A induced higher levels of IgG antibodies against E2.C8A than against E2 aggregates. These results demonstrate that the E2 protein aggregates induced the highest levels of IgG titers irrespective of the tested antigen and E2.C8A protein elicits serum responses that are more specific for E2.C8A monomers. 

Lastly, a HCV vaccine should ideally be pangenotypic. In the present study, both MVA-HCV and E2 vaccines are based on genotype 1a, which is the most prevalent genotype [88]. We previously reported that MVA-HCV also elicited T cell responses against genotype 1b [23] and in the present study we found that sera from mice immunized with the E2/E2 aggregates forms, besides to bind to core-E1-E2 proteins of genotype 1a, also crossreacted to core-E1-E2 proteins of genotypes 2–5. The apparent lack of reactivity by sera from the other groups could be related to the overall lower level of binding antibodies. 

Our immunization regimen using MVA-HCV prime and the single E2 protein boost did not induce NAbs in mice. These results are in line with those reported by others that show that mice do not elicit HCV neutralizing antibodies [89,90]. In contrast, several studies have demonstrated that mice and other small mammals can induce neutralizing antibodies [55,62,91,92]. The experimental conditions between the studies that reported induction of NAbs and ours differed widely so it is therefore difficult to explain this discrepancy. First, most of these studies used significantly higher antigen doses: up to 50 μg E2 per mouse immunization. Second, usually three immunizations are given to the animals, while our regimen only includes two. Third, immunogen forms are different: some use different strains that might be more immunogenic, e.g., HCV-1 [91] or use nanoparticles to increase E2 immunogenicity [73,89]. Fourth, some animals are more capable of inducing NAbs than others: e.g., guinea pigs and rabbits are able to induce neutralizing antibodies against neutralization-resistant HIV-1 strains, while mice usually do not elicit such responses [46,93,94]. Fifth, HCV neutralization assay protocols differ between labs and not all assays seem to take into account the potential issues that might arise from using mouse sera in neutralization assays [46]. Standard procedures, protocols, and controls for performing HCV neutralization assays will be needed to be able to compare neutralization assay results between different labs. 

To induce neutralizing antibodies using our immunization protocol, additional boosts with E2 protein will probably be needed. Furthermore, using defined nanoparticles that properly present E2 [73] might improve the (humoral) immune responses compared to the undefined E2 aggregates used in this study. Lastly, a stabilized native-like mimic of the complete E1E2 complex might be necessary for generating NAbs that effectively recognize neutralizing epitopes that are only available on well-folded native E1E2 on the virion [44,93,95]. Generating a homogeneous soluble E1E2 complex might benefit from taking into account our findings on which cysteines can be removed without adverse effects on antigenicity. Furthermore, the use of replicons in combination with MVA-HCV [25] might be another way to further enhance T cell immune responses to HCV that might also help to improve the quality of the humoral response.

In summary, our study indicates that an immunization protocol consisting of an immunogen focused on T cell activation (MVA) and a protein boost focused on activating B cells (E2) induces a more balanced immune response than a homologous strategy consisting of the same prime and boost. These results will inform optimized vaccine strategies using a similar MVA prime to induce broad CD4 T cell response but probably will need more optimized E2 designs to induce a higher quality B cell response. 

## 5. Conclusions

The present study demonstrates that combining MVA-HCV with optimized HCV E2 glycoproteins more efficiently engage the HCV-specific T and B cell arms of the immune system and this will inform the design of promising vaccine strategies aimed at inducing a protective response. 

## Supplementary Materials

The following are available online at https://www.mdpi.com/2076-393X/8/3/440/s1, Figure S1: Unedited and non-cropped Western Blot, Figure S2: Unedited and non-cropped Coomassie gels.
