# Supplementary material for: Optimized Hepatitis C Virus (HCV) E2 Glycoproteins and their Immunogenicity in Combination with MVA-HCV
Source: Vaccines (Basel). 2020 Aug 5;8(3):440. doi: 10.3390/vaccines8030440 (PMC7563715; doi:10.3390/vaccines8030440)
Supplement: Supplementary file 1 [file vaccines-08-00440-s001.pdf]

Supplementary Materials

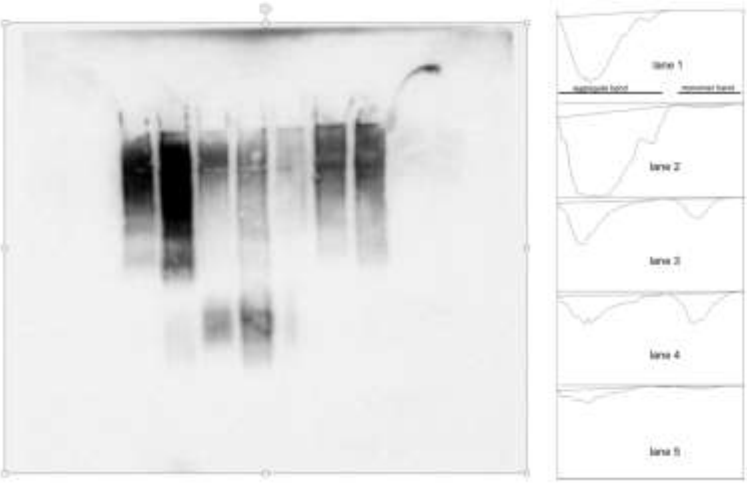

Area under the curve

lanes	band 1 (aggregate)	band 2 (monomer)
1 (E2_MPER)	39398.2	0.0
2 (E2)	71364.3	1144.9
3 (E2_MPER.C8A)	17243.8	5757.1
4 (E2.C8A)	14974.1	10348.5
5 (mock)	6403.0	820.3

Figure S1. Unedited and non-cropped Western Blot.

A

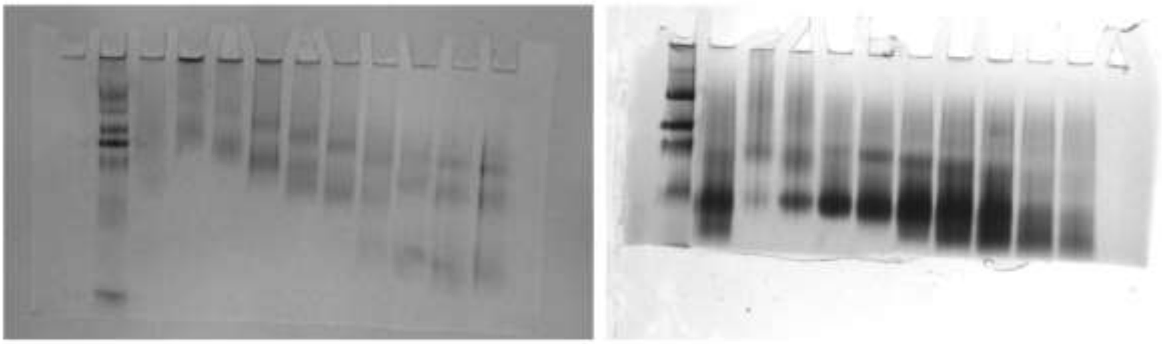

B

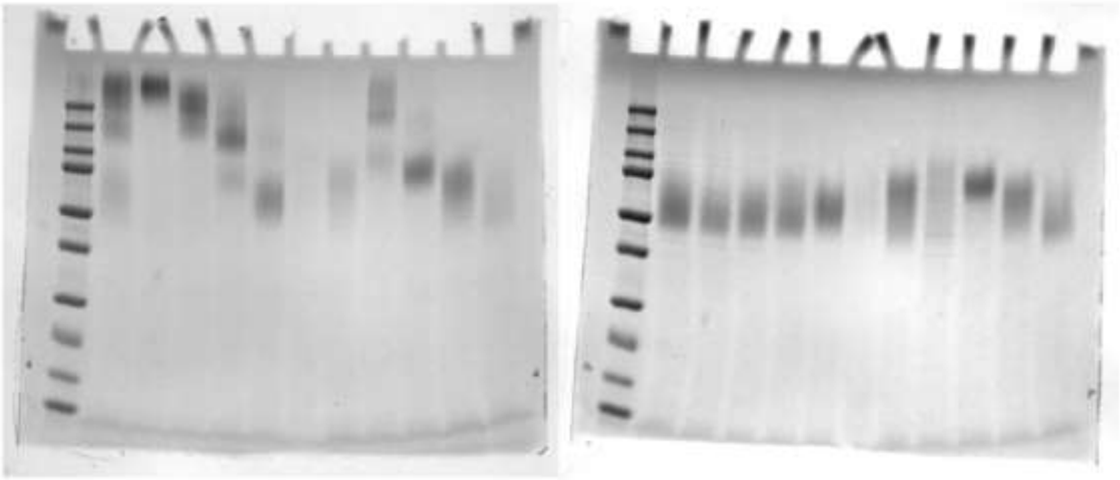

Figure S2. Unedited and non-cropped Coomassie gels.
